# Can fund shareholding inhibit insufficient R&D input?——Empirical evidence from Chinese listed companies

**DOI:** 10.1371/journal.pone.0248674

**Published:** 2021-03-25

**Authors:** Xiao Li, Gang Liu

**Affiliations:** 1 Systems and Industrial Engineering Technology Research Center, Zhongyuan University of Technology, Zhengzhou, Henan, China; 2 School of Business Administration, Hubei University of Economics, Wuhan, Hubei, China; Shandong University of Science and Technology, CHINA

## Abstract

Based on the principal-agent theory and the financial management theory, this study analyzes the impact of fund shareholding on corporate insufficient R&D input, and explores the action mechanism of fund shareholding on corporate innovation activities. The results show that fund shareholding is helpful to inhibit the insufficient R&D input. Moreover, this inhibiting effect is mainly reflected in the case of higher risk of financial failure. The further analyses show that the higher level of marketization strengthens the inhibiting effect of fund shareholding on insufficient R&D input. Finally, it is suggested that fund companies should be encouraged to hold shares of listed companies, and the proposal power of fund companies in the shareholders’ meeting should be appropriately enhanced. And it is suggested that the regulators continue to promote the development of securities investment funds, and guide fund shareholding to play an active role in external governance. Also, it is suggested that the regulators promote the process of regional marketization, to strengthen the positive effect of fund shareholding on innovation activities.

## 1. Introduction

Innovation is a primary driving force for development and the strategic underpinning for building a modern economic system. Innovation leads development, and continuous R&D input is an important guarantee for enterprises to carry out efficient innovation. However, the technological innovation is characterized by investment specificity, uncertainty and unpredictability [1]. Corporate innovation is a long-term and highly uncertain activity, which takes a long time and a large amount of capital, and it is difficult to gain profits in a short time [2]. The “rational” manager carries forward innovation activities only if the private benefits of these activities exceed the expected private costs. Based on the principal-agent theory, the management is directly responsible for the operation and management, and some innovative projects that are beneficial to the company but not to individuals may be abandoned by the managers, thus causing agency problems. Moreover, R&D business often requires long-term resource input, and the resulting income is lagging behind, which will lead to insufficient R&D input by the management [3]. Corporate R&D investment is an important guarantee to form long-term competitiveness, and R&D input depends on the game equilibrium between managers and institutional investors [4].

Fund shareholding is an important part of institutional investors’ shareholding. In China, the influence of fund industry in the securities market is increasing. The shareholding ratio of securities investment funds accounts for more than two thirds among all institutional investors, making them the most important institutional investors [5]. However, scholars have not reached a consensus on the relationship between fund shareholding and R&D input. Yan et al. (2020) [6] believed that the increase of fund shareholding ratio is conducive to promoting corporate innovation activities. Fund shareholding can increase R&D input significantly [7]. In contrast, Jiang et al. (2014) [8] found that fund shareholding had no significant promoting effect on R&D input. Wen and Feng (2012) [9], Lai and Sun (2017) [10] believed that fund shareholding had a significant and negative impact on R&D input. Therefore, it is necessary to clarify the relationship between fund shareholding and R&D input at the present stage, to provide some useful recommendations for improving the efficiency of innovation activities.

**Fig 1:**
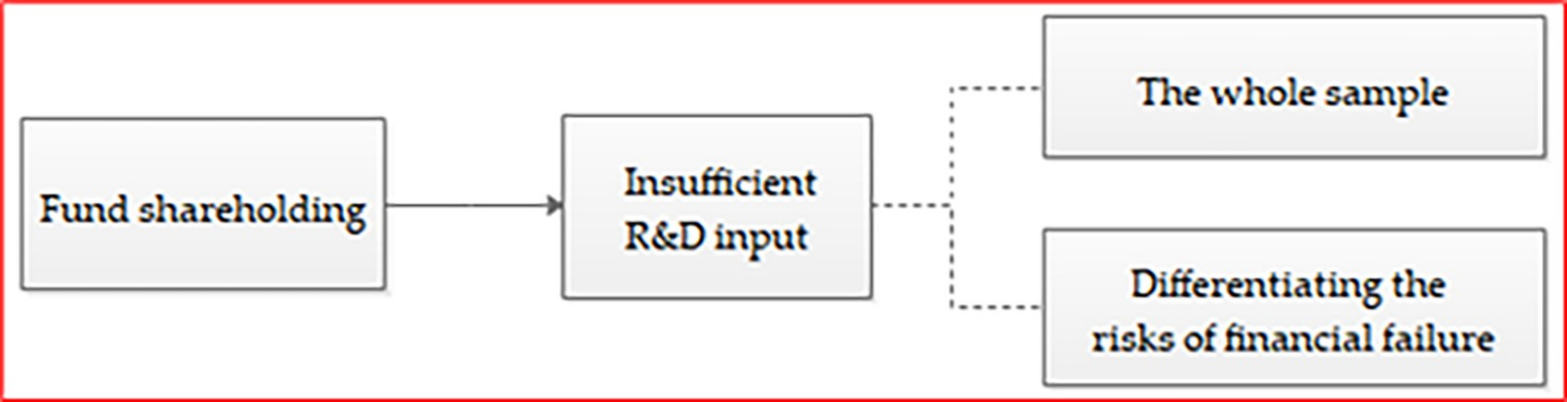
The overall research idea.

Corporate R&D investment is not a rigid project [11]. R&D investment will be affected by many factors, and enterprises are likely to reduce R&D input when they are in financial distress. In accordance with the financial management theory, the risks faced by enterprises mainly include the operational risk and the financial risk. The excessive operating and financial risks will increase the possibility of financial distress, and ultimately aggravate the risk of financial failure. The higher risk of financial failure leads to the low efficiency of resource allocation, resulting in insufficient R&D input and affecting enterprises’ sustainable development. However, the existing literatures have not yet included fund shareholding, R&D input and financial failure risk into the same framework, to explore the mechanism of fund shareholding affecting corporate innovation. Based on this consideration, this study distinguishes the different levels of financial failure risk, and explores the possible mechanism of fund shareholding on insufficient R&D input. Fig 1 shows the overall research idea. The remaining parts are organized as follows. Section 2 provides the literature review, theoretical analysis and research hypothesis. Section 3 presents the data source, variable definition and model setting. Section 4 discusses the descriptive statistics, univariate analysis and variable correlation. Section 5 conducts the model regression analysis. Section 6 carries out the robustness test. Section 7conducts the further analysis. And section 8 draws conclusions and provides recommendations.

The possible contributions are as follows. (1) Different from the existing literatures which take the number of patents as the proxy of corporate innovation activities [12, 13],from the perspective of insufficient R&D input, this study analyzes the impact of fund shareholding on innovation activities, which enriches the research horizons on corporate innovation and the literatures on the economic effects of fund shareholding. (2) Previous scholars studied the influence of fund shareholding on R&D input based on the sample of high-tech enterprises [8], and studied the effect of financing frictions on R&D input [14]. However, there are few literatures to explain the impact of fund shareholding on R&D input from the perspective of financial failure risk. Based on the financial management theory, on basis of distinguishing the risks of financial failure, this study explores the mechanism of fund shareholding affecting corporate innovation activities, and makes up for the shortage that few literatures have included fund shareholding, R&D input and financial failure risk into the same framework. (3) Song et al. (2012) [15] explained the influence of corporate geographical characteristics on institutional investors’ shareholding decisions. Li and Yan (2019) [16] studied the moderating effect of the regional marketization between the additional R&D input caused by venture capital and innovation output. Based on the research of Song et al. (2012) [15],Li and Yan (2019) [16], this study analyzes the moderating effect of the regional marketization between fund shareholding and insufficient R&D input, which has certain practical value for the regulators to promote the marketization process.

## 2. Literature review, theoretical basis, and research hypothesis

### 2.1 Fund shareholding and insufficient R&D input

Institutional investors have improved corporate information disclosure [17], enhanced the sensitivity between the general manager turnover and corporate performance [18]. Institutional shareholders have the opportunity to conduct field research and learn corporate innovation deeply, to promote the management to invest in valuable innovation projects [19]. The active institutional investors can promote enterprises to increase R&D input and drive corporate innovation [20, 21]. As the leading force of institutional investors, securities investment funds actively participate in corporate governance, supervise the management and restrict the behavior of major shareholders, and exert governance effect to alleviate agency conflicts [22–24], and reduce the agency costs [25]. The supervision intensity of institutional investors on enterprises is affected by their investment proportion. When the shareholding ratio is lower, the supervision intensity tends to decrease [26]. In contrast, the increase of funds shareholding ratio is conducive to improving the degree of benefit from corporate earnings, increasing the difficulty of withdrawing investment before the release of negative news, reducing the willingness to “vote with feet”, which is conducive to promoting corporate governance. Corporate governance is the institutional basis of innovation activities, and plays an important role in innovation input [1].

It is the basic path to improve R&D efficiency and promote the steady development of enterprises to alleviate the principal-agent problem by optimizing corporate governance structure. R&D input is the foundation of corporate innovation and the driving force of economic growth. However, R&D activities are characterized by the uncertainty of output, lag and spillover of returns, etc. [27], leading to some serious principal-agent problems. In recent years, although Chinese enterprises’ R&D input has increased, the intensity of R&D input is still significantly lower than that of developed countries in Europe and America [28]. As a result, enterprises’ R&D input often fails to meet the social expectations, and it is difficult to meet the needs of economic development. Based on the principal-agent theory, the agency conflict between owners and managers and that between controlling and minority shareholders are the core contents of corporate governance. In general, securities investment funds tend to hold a large proportion of shares, have an incentive to monitor corporate performance and act as “patient” owners with long-term requirements for improving corporate governance. Fund shareholding plays an important role in improving corporate governance. The allocation of rights, responsibilities and benefits in the process of technological innovation is completed within the framework of corporate governance [29]. The effective governance mechanism is an important factor to promote technological innovation [30]. The good governance mechanism encourages enterprises to pursue both the short-term and long-term goals, which is conducive to the establishment of a long-term input mechanism for technological innovation [31]. Fund companies benefit more from participating in governance than from “voting with feet”. Therefore, they supervise the management to restrain the insufficient R&D input. Securities investment funds collect the investment of retail investors, and have greater influence than individual investors. They are more able to obtain profits through supervision, and thus have a stronger motivation to inhibit the insufficient R&D input. Fund investors supervise the management to promote enterprises to increase R&D investment [32]. Moreover, the positive impact of fund shareholding on listed companies is reflected in its restriction on the encroachment of controlling shareholders [33]. As the agent of medium and small shareholders, fund companies can act as the independent buyers and have the right to speak, to produce the checks and balances, curb the interest encroachment of major shareholders, and avoid the insufficient R&D input. In short, compared with pressure-sensitive institutional investors, which have a business relationship with invested enterprises, securities investment funds are pressure-resistant institutional investors [34]. By participating in governance activities, they can alleviate the information asymmetry, reduce the agency costs, and inhibit the control of R&D capital by the management or the encroachment of the controlling shareholders, to promote the long-term R&D innovation. Thus, different from the interpretation of Zhang et al. (2017) [35] that capital market performance pressure leads to the insufficient R&D input, and the correction effect of investor sentiment on insufficient R&D input described by Xu et al. (2018) [28], this study forms the action path of fund companies participating in corporate governance, alleviating agency problems, and thereby inhibiting the insufficient R&D input.

Based on the above analyses, the following research hypothesis is proposed.

#### 2.1.1 Hypothesis 1

Fund shareholding is conducive to significantly inhibit insufficient R&D input.

### 2.2 Risk of financial failure, fund shareholding and insufficient R&D input

In accordance with the financial management theory, the operational risk mainly comes from the uncertainty of objective economic environment, such as the changes in economic situation and operating environment, the changes in market supply, demand and price, the adjustment of tax policies and financial policies and other external factors, which are difficult to be effectively controlled. The financial risk mainly comes from the proportion of corporate liabilities and the changes in interest rate, which have a great impact on R&D input. The excessive operational and financial risks increase the risk of financial failure. Compared with the lower risk of financial failure, when the risk of financial failure is aggravated, the profitability, solvency and liquidity of assets tend to decrease. And R&D input is more susceptible to financing frictions than physical investment [14]. Furthermore, the higher risk of financial failure tends to lead to inefficient resource allocation, and the lack of sufficient R&D input poses a threat to corporate sustainable development. And R&D business is a long-term innovative activity, which needs more R&D resources. Corporate R&D input is the basic guarantee for developing intangible assets, implementing differentiation strategy and product innovation. Through continuous R&D input, enterprises can form intangible assets such as patents and improve the value of various elements in the portfolio [36]. Without sufficient resource support, it is difficult to succeed in innovation activities [37]. In this case, as “informed traders” with information advantage, fund companies have a better understanding of corporate fundamentals and other information than ordinary investors in the market, and can effectively evaluate corporate value, thus promoting enterprises to carry out valuable investments [38, 39].

Securities investment funds are regarded as more professional and rational institutional investors. Fund companies have professional personnel and operation mode to learn listed companies, have more investment experience and information channels, and pay more attention to corporate long-term value [40]. And R&D business is a continuous accumulation process. Only when R&D capital is accumulated to a certain extent, can it reflect the promoting effect of R&D input on corporate long-term value. In the case of lower risk of financial failure, the enterprise takes the good profitability, debt paying ability and asset liquidity, and has the R&D resources needed to achieve the long-term development. However, when the risk of financial failure is intensified, the top management team’s attention to monetary objectives is negatively related to the R&D input [41]. And then, the management will inevitably neglect the necessary R&D input, which will have a negative impact on corporate long-term value creation. And the aggravation of financial failure risk will be highly concerned by securities investment funds as “informed traders”. Fund investors communicate and cooperate with each other to effectively play the function of corporate governance [42, 43]. By exercising the rights to vote and make proposals, establishing investor alliances, soliciting entrustment voting rights, making public suggestions, communicating with the management, etc. [44], securities investment funds exert the supervision and governance effect [45], and promote the scientificity and rationality of corporate decision-making with a specific monitoring mechanism [46]. Securities investment funds have the motivation and ability to promote enterprises to increase R&D input, choose the innovation project reasonably to enhance corporate value. The spending on R&D is not seen as a cost per se, but rather as an investment [47], which has a profound impact on product production, technology development and other links, and is the material basis for the transformation of technological innovation capacity into practical competitiveness [48]. The increase of R&D input is the manifestation of long-term strategy, to enhance the long-term value and protect the rights and interests of stakeholders. Therefore, fund shareholding is more helpful to restrain the insufficient R&D input, which is more likely to occur when the risk of financial failure is higher.

Based on the above analyses, the following research hypothesis is proposed.

#### 2.2.1 Hypothesis 2

Fund shareholding is more helpful to inhibit insufficient R&D input when the risk of financial failure is aggravated.

## 3. Data source, variable definition and model setting

### 3.1 Data source

From 2011 to 2018, the listed companies publicly traded in Shanghai and Shenzhen stock markets are selected as the sample. The data are from CSMAR China Stock Market Research database and Wind Information financial terminal. This study follows the following principles to screen the data. (1) In view of the particularity of accounting, financial enterprises are excluded. (2) To ensure the integrity and reliability, the observations with missing values and outliers are eliminated. (3) The enterprises processed by ST, * ST during the research period are excluded from the sample. Finally, the financial data of 820 sample enterprises are obtained as the effective observations. To mitigate the effects of extreme data, all continuous variables are Wionsorize treated with bidirectional 1% quantiles.

### 3.2 Variable definition

#### 3.2.1 Explained variable

For the explained variable—Insufficient R&D input (UnderR&D), this study adopts the intensity of corporate annual R&D input to represent R&D input. Meanwhile, with reference to the research of Richardson (2006) [49] and Zhang et al. (2017) [35], corporate R&D input in each year is divided into two parts: (1) Expected R&D input and (2) Unexpected R&D input. The expected R&D input is estimated by the following model 1. Table 1 presents the variable name and description for model 1.

**Table 1:** Variable name and description for model 1.

Symbol	Name	Calculation method
R&D	The intensity of R&D input	R&D input/operating income
GROWTH	Sales growth rate	(Current sales revenue—previous sales revenue)/previous sales revenue
LEV	Asset-liability ratio	Total liabilities/total assets
CASH	Operational cash flow	Net operating cash flow/average total assets. The average total assets are the average of total assets at the beginning and the end. The same below.
AGE	Listed years	The number of years since IPO
LnASSET	Enterprise scale	The natural logarithm of total assets at the beginning
YEAR	Year	The annual effect
IND	Industry	The industry effect. According to the “Guidance on Industry Classification of Listed Companies”(2012 revision) issued by China Securities Regulatory Commission, 16 industries are involved by category and 15 industry dummy variables are set up.
ε		Random disturbance term

**Model 1**.

$${R\&D}_{i,t}=\alpha_{0}+\alpha_{1}{\mathrm{GROWTH}}_{i,t‐1}+\alpha_{2}{\mathrm{LEV}}_{i,t‐1}+\alpha_{3}{\mathrm{CASH}}_{i,t‐1}+\alpha_{4}{\mathrm{AGE}}_{i,t‐1}+\alpha_{5}{\mathrm{LnASSET}}_{i,t}+\alpha_{6}{R\&D}_{i,t‐1}+\alpha_{7}\sum_{t}\mathrm{YEAR}+\alpha_{8}\sum_{t}\mathrm{IND}+\varepsilon_{i,t}$$ (1)

System GMM method is firstly used for regression, and the hypothesis that “all instrumental variables are valid” is rejected (*p*< 0.05), which means that the premise of Systematic GMM is not met. Therefore, model 1 is regressed by fixed effect, to mitigate the adverse effects caused by possible missing variables. Table 2 presents the regression results for model 1. The expected R&D input in year *t* can be obtained by substituting each coefficient into model 1. Then, the actual R&D input is subtracted from the expected R&D input to calculate the residuals. The positive residuals indicate that the actual R&D input is greater than the expected R&D input, meaning that the R&D input is relatively sufficient. Enterprises’ adequate R&D investment is not only conducive to carrying out differentiated R&D projects and forming specialized technologies, but also improves the ability to resist risks in the process of innovation. The increase of R&D input is an important factor to improve innovation efficiency [50, 51]. Different resource allocation will lead to differentiated innovation performance [52]. Enterprises’ technological innovation is a systematic project, which requires a large amount of capital to carry out R&D activities.

**Table 2:** Regression results for model 1.

Variable	Coef. (S.E.)	Variable	Coef. (S.E.)		
Intercept	-3.982(3.276)	L.AGE	0.326***(0.076)	Adj_R^2^	0.126
L.GROWTH	-0.002**(0.001)	LnASSET	0.301**(0.140)	# of obs.	8952
L.LEV	-0.018***(0.003)	L.R&D	0.332***(0.050)		
L.CASH	-0.625***(0.225)	YEAR/IND	YES		

Note

*** Significant at 1%

** Significant at 5%

* Significant at 10%.

Robust standard errors in brackets are clustered at corporate level.

However, the negative residuals mean that the actual R&D input is less than the expected R&D input, and the investment in innovation activities is insufficient, which is difficult to guarantee the expected capital demand of R&D activities, and is not conducive to stimulating the vitality to continuously improve innovation performance. Therefore, the negative residuals represent the insufficient R&D input. To facilitate the regression analyses, the absolute values of negative residuals are adopted to represent UnderR&D.

#### 3.2.2 Explanatory variable

For the measurement of fund shareholding ratio (FUND), since non-tradable shares do not participate in the circulation in the secondary market, this study adopts the percentage of the total shares held by securities investment funds in the company’s tradable A shares as the measurement of FUND. This study makes an average on FUND disclosed in the semi-annual and annual reports, to measure the annual shareholding status of securities investment funds and smooth the adverse impact of different shareholding duration on the results. The higher the value of this indicator, the higher the average shareholding of securities investment funds in the current period.

#### 3.2.3 Control variable

With reference to the research of Bird and Karolyi (2016) [17], Zhang et al. (2017) [35], this study takes Return on assets (ROA), Asset turnover ratio (TAT), Asset-liability ratio (LEV), Sales growth rate (GROWTH), Ownership concentration (ShrZ), Listed years (AGE), The proportion of independent directors (IN_DIREC), Enterprise scale (LnASSET), Executive compensation (LnSALARY), Audit opinion (AUDIT) and Property attribute (STATE) as the control variables, to investigate their possible effects on UnderR&D, respectively. Also, the annual effect (YEAR) and industry effect (IND) are controlled in the regression analyses. Table 3 shows the variable name and description in model 2 below.

**Table 3:** Variable name and description for model 2.

Nature	Symbol	Name	Calculation method
Explained variable	UnderR&D	Insufficient R&D input	From model 1.
Explanatory variable	FUND	Fund shareholding ratio	Total shares held securities investment funds /the company’s tradable A shares
Control Variable	ROA	Return on assets	Net profit/average total assets
	TAT	Asset turnover ratio	Current operating income/average total assets
	LEV	Asset-liability ratio	The same as in model 1
	GROWTH	Sales growth rate	The same as in model 1
	ShrZ	Ownership concentration	Shareholding ratio of the largest shareholder / that of the second largest shareholder
	AGE	Listed years	The same as in model 1
	IN_DIREC	The proportion of independent directors	The proportion of independent directors in the board of directors
	LnASSET	Enterprise scale	The same as in model 1
	LnSALARY	Executive compensation	The natural logarithm of the total annual salaries of directors, supervisors and executives
	AUDIT	Audit opinion	A dummy variable set according to the audit opinion. Where, the value of standard unreserved opinion is 1; otherwise, it is 0.
	STATE	Property attribute	Dummy variable, 1 for state-owned enterprises; otherwise, it’s 0
	YEAR	Year	The same as in model 1
	IND	Indusry	The same as in model 1

### 3.3 Model setting-up

With reference to the research of Bird and Karolyi (2016) [17], Zhang et al. (2017) [35], the following model 2 is constructed. Where, to alleviate the endogeneitycaused by reverse causality, the control variables—ROA, TAT, LEV, GROWTH, LnSALARY and AUDIT are taken as the first-order lag in the regression. On this basis, the regression is conducted for model 2 based on the whole sample to test hypothesis 1 above.

**Model 2**.

$${\mathrm{UnderR}\&D}_{i,t}=\beta_{0}+\beta_{1}{\mathrm{FUND}}_{i,t}+\beta_{2}{\mathrm{ROA}}_{i,t‐1}+\beta_{3}{\mathrm{TAT}}_{i,t‐1}+\beta_{4}{\mathrm{LEV}}_{i,t‐1}+\beta_{5}{\mathrm{GROWTH}}_{\mathrm{it}‐1}+\beta_{6}{\mathrm{ShrZ}}_{\mathrm{it}}+\beta_{7}{\mathrm{AGE}}_{\mathrm{it}}+\beta_{8}\mathrm{IN}\_{\mathrm{DIREC}}_{i,t}+\beta_{9}{\mathrm{LnASSET}}_{i,t}+\beta_{10}\mathrm{LnSALAR}Y_{i,t‐1}+\beta_{11}{\mathrm{AUDIT}}_{i,t‐1}+\beta_{12}{\mathrm{STATE}}_{i,t}+\beta_{13}\sum_{t}\mathrm{YEAR}+\beta_{14}\sum_{t}\mathrm{IND}+\varepsilon_{i,t}$$ (2)

Meanwhile, in order to clarify the possible mechanism of fund shareholding on insufficient R&D input, this study distinguishes the risks of financial failure. The possibility of financial failure depends on the company’s comprehensive financial position such as profitability, asset liquidity and financial leverage [53]. Altman (1968) [54] used a model containing multiple variables to analyze financial risks and established a linear and discriminant Z-score model, which began to transform the univariate evaluation into the comprehensive evaluation of financial risks. The Z-Score model comprehensively reflects the basic financial characteristics of an enterprise from the profitability, debt repayment and asset liquidity and so on. Therefore, with reference to the research of Zhu et al. (2014) [55], this study adopts the Z-score model to evaluate the risks of financial failure. The specific calculation of Z-score model is shown in formula (3). Where, Z_1_ represents the ratio of working capital to total assets, which reflects the characteristics of liquidity and size. And the working capital is equal to the difference between current assets and current liabilities. The more the working capital, the smaller the risk of debt repayment, which reflects the company’s short-term solvency. Z_2_represents the ratio of retained earnings to total assets, which measures the accumulated profits and reflects the company’s operating period. Z_3_ represents the ratio of earnings before interest and tax to total assets, which measures the productive capacity of the company’s assets without considering the effects of taxes and financing. It is a measure of the profitability achieved by the company using creditors’ equities and shareholders’ equities. The higher this ratio, the better the utilization of assets, and the higher the level of operation and management. And Z_4_ represents the ratio of total market value to total liabilities, which measures the possible decline of equity value before insolvency, reflects the relative relationship between equity capital and debt capital, and indicates whether the basic financial structure is stable. Also, this ratio reflects the impact of the capital market prosperity on the guarantee degree of debt capital from shareholder capital under a specific macroeconomic background. Besides, Z_5_ represents the ratio of operating income to total assets, which measures how efficiently the company uses its assets to generate sales. When the macro economy is running well and the product trade market is more active, enterprises will have a good effect in increasing income.

$$Z‐\mathrm{score}=1.200\times Z_{1}+1.400\times Z_{2}+3.300\times Z_{3}+0.600\times Z_{4}+0.999\times Z_{5}$$ (3)

According to the research of Altman (1968) [54] and Zhu et al. (2014) [55], in general, when Z-score is lower, the likelihood of financial failure tends to increase. And *Z-score*< 1.810 indicates that the bankruptcy crisis is lurking within the enterprise. When Z-score is higher, corporate current financial performance is relatively stable. *Z-score*> 2.675 means that corporate financial performance is good and the possibility of bankruptcy is low. And 1.810 ≤ *Z-score* ≤ 2.675 indicates that the financial performance is extremely unstable, which is known as the “gray zone”. Based on the consideration of “prudence”, this study believes that *Z-score*> 2.675 indicates a lower risk of financial failure. *Z-score* ≤ 2.675 indicates a higher risk of financial failure. On this basis, the regression analyses are carried out for model 2 based on the sample with *Z-score*> 2.675 and that with *Z-score* ≤ 2.675, which are adopted to test hypothesis 2 above.

## 4. Descriptive statistics, univariate analysis and variable correlation

### 4.1 Descriptive statistics

Table 4 reports the descriptive statistics for model 2. Where, for the explained variable, the mean (standard deviation) of UnderR&D is 2.822 (1.998), indicating that there is an objective situation of insufficient R&D input in the enterprises, and the insufficient R&D input has a certain degree of difference. The insufficient R&D input means the lack of innovation awareness, and the establishment of innovation-driven economy has a long way to go. For the explanatory variable, the maximum of FUND is 42.48%, and its mean (median) is 5.07% (2.84%), implying that the fund shareholding ratio has a large span among different enterprises, which may be caused by the fund shareholding’ industry preferences.

**Table 4:** Descriptive statistics.

Variable	Mean	Median	Maximum	Minimum	Deviation	Observations
UnderR&D	2.822	2.519	17.048	0.000	1.998	3594
FUND	5.069	2.836	42.480	0.015	6.358	3594
ROA	6.317	5.361	25.552	-13.911	5.746	3594
TAT	0.757	0.660	2.407	0.117	0.453	3594
LEV	46.887	47.535	82.876	4.901	18.612	3594
GROWTH	12.964	8.827	134.295	-41.913	26.930	3594
ShrZ	10.950	4.550	91.693	1.000	16.391	3594
AGE	13.548	14.000	28.000	1.000	5.835	3594
IN_DIREC	0.370	0.333	0.571	0.333	0.053	3594
LnASSET	22.916	22.764	26.091	20.277	1.207	3594
LnSALARY	15.485	15.439	17.234	13.823	0.703	3594
AUDIT	0.984	1.000	1.000	0.000	0.124	3594
STATE	0.502	1.000	1.000	0.000	0.500	3594

In the control variables, the mean (minimum) of ROA is 6.32% (-13.91%), implying that the overall returns on assets needs to be further improved. The minimum of GROWTH is -41.91%, showing that the development of some enterprises is more worrying. And the mean (standard deviation) of LnASSET is 22.916 (1.207). The mean (standard deviation) of TAT is 0.757 (0.453). There are significant differences in the enterprises’ size. And the asset turnover speed of different enterprises presents a large difference. The mean of LEV is 46.89%, implying that the capital structure is maintained at a reasonable level. Besides, the mean of ShrZ is 10.950, suggesting that the degree of equity concentration is higher. The longest listed age is 28 years, and the shortest is less than 1 year, which is in line with IPO current status in Chinese capital market. Also, the minimum of IN_DIREC is 0.333, which is in line with the regulation that “the board members of a listed company shall have at least one third of independent directors”, issued by China Securities Regulatory Commission. Meanwhile, the executive compensation varies to a certain extent. On average, the proportion of central and local state-owned enterprises accounts for50.20%. The mean of AUDIT is 0.984, indicating that the majority of listed enterprises have acquired the standard audit reports, which ensure the reliability of the data used in this paper.

### 4.2 Univariate analysis

The sample with *Z-score*> 2.675 and that with *Z-score* ≤ 2.675 are grouped. For these two subsamples, the tests on mean and median are conducted to compare the variables’ differences in model 2.

As shown in Table 5, in the sample with *Z-score* ≤ 2.675, the mean and median of UnderR&D are higher than those in that with *Z-score*> 2.675, which are significant at 1%, indicating that the insufficient R&D input is more likely to occur when the risk of financial failure is higher. Meanwhile, in the sample with *Z-score*> 2.675, the mean and median of FUND are higher than those in that with *Z-score* ≤ 2.675, which are significant at 1%, indicating that there is an obvious difference in the shareholding ratio of fund companies in both sets of observations.

**Table 5:** Univariate analysis for *Z-score*> 2.675 and *Z-score*≤ 2.675.

Variable	Mean difference			Median difference		
	*Z-score* > 2.675	*Z-score* ≤ 2.675	Mean Diff	*Z-score* > 2.675	*Z-score* ≤ 2.675	Chi^2^
UnderR&D	2.577	3.139	-0.562***	2.099	3.077	70.709***
FUND	6.329	3.443	2.886***	3.675	2.006	138.589***
ROA	8.272	3.791	4.481***	7.568	3.872	454.748***
TAT	0.853	0.632	0.221***	0.722	0.579	120.234***
LEV	35.729	61.306	-25.577***	35.088	62.123	1498.467***
GROWTH	14.941	10.410	4.531***	10.197	7.127	16.291***
ShrZ	9.921	12.280	-2.359***	4.510	4.610	0.280
AGE	12.769	14.555	-1.785***	13.000	15.000	39.106***
IN_DIREC	0.369	0.372	-0.003*	0.333	0.333	2.921*
LnASSET	22.381	23.606	-1.225***	22.284	23.445	749.620***
LnSALARY	15.437	15.546	-0.108***	15.391	15.506	12.237***
AUDIT	0.991	0.976	0.015***	1.000	1.000	—
STATE	0.402	0.631	-0.229***	0.000	1.000	—
# of obs.	2026	1568		2026	1568	

Note

*** Significant at 1%

** Significant at 5%

* Significant at 10%. The mean and median differences are tested by T test and Non-parametric 2-sample test, respectively.

Besides, for most control variables, the differences in the mean and median are significant statistically (*p* < 0.01 or *p* < 0.10) in the two subsamples. The above results indicate that it is necessary to distinguish the risk levels of financial failure, to further explore the mechanism of fund shareholding on insufficient R&D input.

### 4.3 Variable correlation

Table 6 reports the correlation of variables in model 2. Where, FUND is negatively and significantly correlated with UnderR&D (-0.070, *p <*0.01). This result indicates preliminarily that fund shareholding changes the managers’ short-sighted and opportunistic behaviors, makes enterprises pay more attention to the long-term orientation, increase the input in technological innovation, and avoid the insufficient R&D input. Securities investment funds have advantages in scale, personnel and information, so that fund shareholding is positively correlated with R&D expenditure [56].

**Table 6:** The correlation between variables.

Variable	UnderR&D	FUND	ROA	TAT	LEV	GROWTH	ShrZ	AGE	IN_DIREC	LnASSET	LnSALARY	AUDIT
UnderR&D	1.000											
FUND	-0.070***	1.000										
ROA	0.019	0.342***	1.000									
TAT	0.056***	0.116***	0.179***	1.000								
LEV	0.150***	-0.101***	-0.357***	0.070***	1.000							
GROWTH	-0.134***	0.181***	0.228***	0.103***	-0.010	1.000						
ShrZ	0.154***	-0.130***	-0.061***	0.051***	0.074***	-0.110***	1.000					
AGE	0.755***	-0.074***	0.004	-0.047***	0.161***	-0.146***	0.097***	1.000				
IN_DIREC	-0.001	-0.023	-0.023	-0.037**	0.007	-0.014	0.005	-0.033**	1.000			
LnASSET	0.318***	-0.070***	-0.028*	-0.017	0.533***	-0.024	0.049***	0.264***	0.117***	1.000		
LnSALARY	0.131***	0.068***	0.208***	0.075***	0.136***	0.062***	-0.142***	0.210***	-0.007	0.451***	1.000	
AUDIT	-0.019	0.048***	0.165***	0.048***	-0.064***	0.072***	-0.034**	-0.005	-0.018	0.012	0.073***	1.000
STATE	0.383***	-0.106***	-0.134***	0.009	0.244***	-0.185***	0.240***	0.385***	0.024	0.327***	0.036**	0.019

Note

*** Significant at 1%

** Significant at 5%

* Significant at 10%.

In the control variables, TAT (0.056), LEV (0.150), ShrZ (0.154), AGE (0.755), LnASSET (0.318), LnSALARY (0.131) and STATE (0.383) are positively and significantly correlated with UnderR&D (*p <*0.01). However, GROWTH is negatively and significantly correlated with UnderR&D (-0.134, *p <*0.01). These results indicate that the selection of control variables is very necessary, and ensure the rationality of model 2. Besides, the maximum correlation between the explanatory variable and control variables, and between the control variables is 0.533, which exists between LEV and LnASSET, less than the threshold value of 0.800, indicating that there is no serious multicollinearity in model 2, and providing a reliable guarantee for subsequent regression.

## 5. Model regression analysis

For the whole sample, the sample with *Z-score*> 2.675 and that with *Z-score* ≤ 2.675, this study carries out the regression respectively. Table 7 reports the regression results for model 2. In the whole sample, the coefficient on FUND is negative and significant (-0.011, *p*< 0.01), indicating that with the increase of fund shareholding, it will promote the power and ability of fund investors to improve corporate R&D input decision. Fund shareholding enables fund investors to pay more attention to the long-term value of investment, actively participate in corporate governance in explicit or implicit ways, and guide enterprises to actively invest in R&D projects which can enhance corporate value, to avoid the insufficient R&D input. Hypothesis 1 above is verified. The above result does not support the conclusion of Wen and Feng (2012) [9], Lai and Sun (2017) [10] that fund shareholding has a negative impact on corporate innovation. And in the sample with *Z-score*> 2.675 and that with *Z-score* ≤ 2.675, the coefficients on FUND are negative and significant (-0.006, *p*< 0.10; -0.025, *p*< 0.01). And by Fisher’s Permutation test, for the sample with *Z-score*> 2.675 and that with *Z-score* ≤ 2.675, the difference in the coefficients on FUND is positive and significant (0.019, *p*< 0.01). Among the enterprises with higher risk of financial failure, fund shareholding is more helpful to restrain the insufficient R&D input. For the enterprises with higher risk of financial failure, fund shareholding is more conducive to improving corporate governance, reducing the implementation risk of R&D activities, and promoting the investment intensity of innovative business. Hypothesis 2 above is verified. It is an important mechanism for fund shareholding to promote corporate innovation to avoid insufficient R&D input when the risk of financial failure is higher.

**Table 7:** Regression results for model 2.

Variable	The whole sample	*Z-score*> 2.675	*Z-score* ≤ 2.675
	Coef. (S.E.)	Coef. (S.E.)	Coef. (S.E.)
Intercept	-6.546*** (0.696)	-5.410*** (0.855)	-6.748*** (0.954)
FUND	-0.011*** (0.003)	-0.006* (0.003)	-0.025*** (0.007)
L.ROA	-0.005 (0.004)	-0.007 (0.005)	0.003 (0.008)
L.TAT	0.533*** (0.041)	0.606*** (0.051)	0.406*** (0.092)
L.LEV	-0.016*** (0.001)	-0.017*** (0.002)	-0.011*** (0.003)
L.GROWTH	-0.073 (0.082)	-0.074 (0.094)	-0.121 (0.149)
ShrZ	0.042 (0.140)	0.097 (0.232)	-0.059 (0.158)
AGE	0.268*** (0.004)	0.255*** (0.006)	0.283*** (0.005)
IN_DIREC	0.084 (0.343)	0.491 (0.483)	-0.299 (0.507)
LnASSET	0.413*** (0.022)	0.418*** (0.036)	0.412*** (0.029)
L.LnSALARY	-0.207*** (0.032)	-0.234*** (0.045)	-0.201*** (0.045)
L.AUDIT	-0.825*** (0.297)	-0.926*** (0.256)	-0.722* (0.426)
STATE	0.044 (0.046)	0.196*** (0.064)	-0.113 (0.070)
YEAR/IND	YES	YES	YES
# of obs.	3594	2026	1568
Adj_R^2^	0.707	0.707	0.704
F_Value	382.44***	158.77***	129.46***

Note

*** Significant at 1%

** Significant at 5%

* Significant at 10%.

Robust standard errors in brackets are clustered at corporate level.

In the control variables, for the whole sample, the sample with *Z-score*> 2.675 and that with *Z-score* ≤ 2.675, the coefficients on L.LEV are negative and significant (-0.016, *p*< 0.01; -0.017, *p*< 0.01; -0.011, *p*< 0.01). And the coefficients on L.AUDIT are negative and significant (-0.825, *p*< 0.01; -0.926, *p*< 0.01; -0.722, *p*< 0.10). The creditors’ governance effect and external auditors’ supervision are helpful to stimulate enterprises’ enthusiasm to carry out R&D activities, and strengthen the intensity of R&D input to avoid the possible under-investment in R&D activities. Also, the coefficients on L.LnSALARY are negative and significant (-0.207, *p*< 0.01; -0.234, *p*< 0.01; -0.201, *p*< 0.01), indicating that executive compensation incentive can promote R&D input [57], and restrain enterprises’ insufficient input in innovation. However, the coefficients on L.TAT are positive and significant (0.533, *p*< 0.01; 0.606, *p*< 0.01; 0.406, *p*< 0.01). The stronger the operating capacity is, the lower the R&D input will be. One possible explanation is that the more well-run enterprises are, the more resources are allocated to the areas where new investment opportunities are directed, thus crowding out R&D input. And the coefficients on AGE are positive and significant (0.268, *p*< 0.01; 0.255, *p*< 0.01; 0.283, *p*< 0.01). The longer an enterprise’s listed period is, the weaker its innovation motivation will be [13, 58], leading to insufficient input in innovation activities. The coefficients on LnASSET are positive and significant (0.413, *p*< 0.01; 0.418, *p*< 0.01; 0.412, *p*< 0.01), suggesting that large-scale enterprises’ internal structure is complex and the business process is tedious, which have a certain degree of negative effect on R&D input. Besides, in the sample with *Z-score*> 2.675, the coefficient on STATE is positive and significant (0.196, *p*< 0.01). In state-owned enterprises, professional managers choose a conservative and progressive R&D mode, to stick to the bottom line of maintaining and increasing the value of state-owned assets and ensure the standardization of capital use. This mode does not absorb external knowledge adequately and attaches little importance to R&D input, so it is difficult to adapt to the rapid changes in the market. The coefficients on the remaining control variables are not significant statistically.

## 6. Robustness test

### 6.1 Re-estimate the explained variable

To avoid the interference caused by the possible systematic deviation of Richardson’s (2006) [49] model in calculating UnderR&D, this study refers to the design of Yang and Li (2018) [59]. The absolute values of negative residuals in model 1 are divided into 10 groups on average. After the elimination of the two groups closest to 0, the remaining groups represent UnderR&D. On this basis, the explained variable in model 2 is re-estimated and the regression analyses are conducted again. Table 8 reports the corresponding results.

**Table 8:** Regression results after re-estimating UnderR&D for model 2.

Variable	The whole sample	*Z-score*> 2.675	*Z-score* ≤ 2.675
	Coef. (S.E.)	Coef. (S.E.)	Coef. (S.E.)
Intercept	-5.068*** (0.753)	-4.825*** (1.030)	-4.726*** (1.042)
FUND	-0.013*** (0.003)	-0.008** (0.004)	-0.027*** (0.007)
L.ROA	-0.001 (0.004)	-0.003 (0.005)	-0.001 (0.009)
L.TAT	0.475*** (0.042)	0.522*** (0.052)	0.312*** (0.095)
L.LEV	-0.014*** (0.001)	-0.016*** (0.002)	-0.010*** (0.003)
L.GROWTH	-0.153* (0.090)	-0.175* (0.101)	-0.151 (0.163)
ShrZ	0.043 (0.121)	0.290 (0.187)	-0.162 (0.151)
AGE	0.257*** (0.004)	0.245*** (0.006)	0.271*** (0.006)
IN_DIREC	0.491 (0.364)	0.916* (0.536)	-0.039 (0.528)
LnASSET	0.376*** (0.022)	0.415*** (0.037)	0.371*** (0.030)
L.LnSALARY	-0.201*** (0.033)	-0.245*** (0.047)	-0.183*** (0.046)
L.AUDIT	-0.770** (0.339)	-0.900*** (0.299)	-0.633 (0.462)
STATE	0.0004 (0.048)	0.066 (0.065)	-0.070 (0.076)
YEAR/IND	YES	YES	YES
# of obs.	2802	1497	1305
Adj_R^2^	0.671	0.681	0.658
F_Value	266.71***	111.11***	87.57***

Note

*** Significant at 1%

** Significant at 5%

* Significant at 10%.

Robust standard errors in brackets are clustered at corporate level.

In the whole sample, the coefficient on FUND is negative and significant (-0.013, *p*< 0.01), indicating that fund shareholding can inhibit the lack of R&D input and supervise the management to strengthen the intensity of R&D input. From this point, fund shareholding is conducive to guiding enterprises to carry out the valuable investment and promote enterprises to form the long-term competitiveness. Hypothesis 1 is verified again. And in the sample with *Z-score*> 2.675 and that with *Z-score* ≤ 2.675, the coefficients on FUND are negative and significant (-0.008, *p*< 0.05; -0.027, *p*< 0.01). After Fisher’s Permutation test, for the sample with *Z-score*> 2.675 and that with *Z-score* ≤ 2.675, the difference in the coefficients on FUND is positive and significant (0.018, *p*< 0.01). Fund shareholding has a more obvious inhibiting effect on insufficient R&D input in the enterprises with higher risk of financial failure. Hypothesis 2 is verified again. In the case of higher risk of financial failure, fund companies strengthen the monitoring of technological innovation, prevent the higher risk of financial failure to produce the inefficient resource allocation, and promote enterprises to carry out innovation activities.

In the control variables, the conclusions on L.TAT, L.LEV, AGE, LnASSET, L.LnSALARY and L.AUDIT are consistent with those from Table 7. Besides, in the whole sample and that with *Z-score*> 2.675, the coefficients on L.GROWTH are negative and significant (-0.153, *p*< 0.10; -0.175, *p*< 0.10), implying that the enterprises with better growth are more willing to increase R&D input to achieve the long-term development. However, in the sample with *Z-score*>2.675, the coefficient on IN_DIREC is positive and significant (0.916, *p*< 0.10), suggesting that the regulators should urge independent directors to exert their governance effect, and supervise enterprises to attach importance to R&D input to adapt to the rapid changes in the market.

### 6.2 Instrumental variable method

Since fund companies may choose target companies with higher growth potential, their shareholding proportion may be determined according to the target companies’ intensity of R&D input. Thus, the endogeneity problem may exist in fund companies’ investment decisions. In order to weaken the adverse effect of the endogeneity on the conclusions, the instrumental variable method is adopted. An appropriate instrumental variable is highly correlated with the possible endogenous variable and not with the explained variable. Obviously, the shareholding ratio of fund companies in the previous period will affect that in the current period, while its influence on the R&D input in the current period is weak. Therefore, with reference to the research of He et al. (2018) [60], in the first-stage regression, the first-order lag of FUND (L.FUND) is introduced. Meanwhile, with reference to the research of Lee and Chung (2015) [61], Zhang and Li (2017) [62],the number of funds holding shares (Num_FUND) is added as the instrumental variable. The higher the number of funds holding shares, the higher their total holding proportion, and the number of funds holding shares is likely to not directly affect the current R&D input of the enterprise. In this paper, the numbers of funds holding shares disclosed in the semi-annual and annual reports are averaged to measure the average number of funds holding shares in the year. Table 9 reports the test results of instrumental variable method for model 2.

**Table 9:** Test results of instrumental variable method for model 2.

Variable	The whole sample		*Z-score*> 2.675		*Z-score* ≤ 2.675	
	Stage 1	Stage 2	Stage 1	Stage 2	Stage 1	Stage 2
	Coef. (S.E.)	Coef. (S.E.)	Coef. (S.E.)	Coef. (S.E.)	Coef. (S.E.)	Coef. (S.E.)
Intercept	32.612***(2.690)	-3.893***(0.737)	38.718***(4.573)	-3.322***(0.956)	23.034***(2.808)	-4.743***(1.023)
FUND		-0.017***(0.004)		-0.006(0.005)		-0.053***(0.010)
L.ROA	-0.058***(0.018)	-0.003(0.004)	-0.055**(0.023)	-0.007(0.005)	-0.086***(0.024)	0.005(0.009)
L.TAT	0.135(0.182)	0.562***(0.042)	-0.022(0.244)	0.631***(0.052)	0.119(0.259)	0.446***(0.094)
L.LEV	0.031***(0.005)	-0.016***(0.001)	0.042***(0.008)	-0.018***(0.002)	0.023***(0.007)	-0.011***(0.003)
L.GROWTH	0.009***(0.003)	-0.097(0.086)	0.012***(0.004)	-0.094(0.097)	0.005(0.003)	-0.013(0.016)
ShrZ	-0.017***(0.003)	0.004(0.141)	-0.024***(0.005)	0.095(0.232)	-0.011***(0.003)	-0.002(0.002)
AGE	-0.002(0.014)	0.268***(0.004)	-0.013(0.023)	0.253***(0.006)	0.020(0.015)	0.285***(0.006)
IN_DIREC	-3.070**(1.335)	0.132(0.347)	-4.068*(2.344)	0.463(0.493)	-3.253***(1.158)	-0.302(0.503)
LnASSET	-1.418***(0.110)	0.414***(0.022)	-1.817***(0.189)	0.426***(0.036)	-0.924***(0.112)	0.416***(0.030)
L.LnSALARY	-0.192*(0.116)	-0.209***(0.033)	-0.041(0.191)	-0.240***(0.046)	-0.236**(0.120)	-0.195***(0.045)
L.AUDIT	0.858**(0.353)	-0.912***(0.330)	1.060(0.679)	-1.045***(0.313)	0.951**(0.473)	-0.764*(0.445)
STATE	0.001(1.559)	0.061(0.047)	0.043(0.235)	0.206***(0.064)	-0.139(0.180)	-0.096(0.073)
L.FUND	0.527***(0.021)		0.541***(0.025)		0.435***(0.033)	
Num_FUND	0.030***(0.003)		0.032***(0.004)		0.026***(0.003)	
YEAR/IND	YES	YES	YES	YES	YES	YES
# of obs.	3465	3465	1957	1957	1508	1508
Adj_R^2^	0.603	0.707	0.608	0.709	0.530	0.705
F_Value	62.55***		67.11***		76.99***	
Wald_chi^2^		11195.75***		11705.07***		24568.80***

Note

*** Significant at 1%

** Significant at 5%

* Significant at 10%.

Robust standard errors in brackets are clustered at corporate level.

As shown in Table 9, for the whole sample, the sample with *Z-score*> 2.675 and that with *Z-score* ≤ 2.675, in the first-stage regression, the coefficients on L.FUND are positive and significant (0.527, *p*< 0.01; 0.541, *p*< 0.01; 0.435, *p*< 0.01), and those on Num_FUND are positive and significant (0.030, *p*< 0.01; 0.032, *p*< 0.01; 0.026, *p*< 0.01). Moreover, the F-statistics of weak instrumental variable test are 571.335, 416.196 and 185.438 respectively, much higher than 10.000, so it is considered that there are no weak instrumental variables [63]. In the second-stage regression, for the whole sample and that with *Z-score* ≤ 2.675, the coefficients on FUND are negative and significant (-0.017, *p*< 0.01; -0.053, *p*< 0.01). However, for the sample with *Z-score*> 2.675, the coefficient on FUND is not statistically significant (-0.006, *p*> 0.10). Once again, the above results show that fund shareholding has an inhibiting effect on the lack of R&D input, and this effect is mainly reflected when the risk of financial failure is higher. Fund shareholding promotes enterprises to significantly increase R&D input based on the principle of maximizing the long-term value [7], and attach importance to R&D input to enhance their sustainable competitiveness. For the control variables, the conclusions on L.TAT, L.LEV, AGE, LnASSET, L.LnSALARY, L.AUDIT and STATE are consistent with those from Table 7 above.

### 6.3 Change the model

In order to further verify the robustness of the above results, based on model 2, this study constructs the following model 3 by adding the interaction item (*FUND×DZ-score*). Where, DZ-score represents the dummy variable for the risk of financial failure (*Z-score*). When the risk of financial failure is higher (*Z-score ≤ 2*.*675*), the value of DZ-score is 1. When the risk of financial failure is lower (*Z-score > 2*.*675*), the value of DZ-score is 0. Meanwhile, for model 3, this study distinguishes two scenarios before and after re-estimating UnderR&D for regression.

Model 3.

$${\mathrm{UnderR}\&D}_{i,t}=\delta_{0}+\delta_{1}{\mathrm{FUND}}_{i,t}+\delta_{2}{\mathrm{DZ}‐\mathrm{score}}_{i,t}+\delta_{3}{\mathrm{FUND}}_{i,t}\times {\mathrm{DZ}‐\mathrm{score}}_{i,t}+\delta_{4}{\mathrm{ROA}}_{i,t‐1}+\delta_{5}{\mathrm{TAT}}_{i,t‐1}+\delta_{6}{\mathrm{LEV}}_{i,t‐1}+\delta_{7}{\mathrm{GROWTH}}_{i,t‐1}+\delta_{8}{\mathrm{ShrZ}}_{i,t}+\delta_{9}{\mathrm{AGE}}_{i,t}+\delta_{10}\mathrm{IN}\_{\mathrm{DIREC}}_{i,t}+\delta_{11}{\mathrm{LnASSET}}_{i,t}+\delta_{12}{\mathrm{LnSALARY}}_{i,t‐1}+\delta_{13}{\mathrm{AUDIT}}_{i,t‐1}+\delta_{14}{\mathrm{STATE}}_{i,t}+\delta_{15}\sum_{t}\mathrm{YEAR}+\delta_{16}\sum_{t}\mathrm{IND}+\varepsilon_{i,t}$$ (4)

In Table 10, columns 1 and 2 report the regression results before re-estimating UnderR&D, and columns 3 and 4 report those after re-estimating UnderR&D. And in columns 2 and 4, to weaken the adverse effect of multicollinearity on the results, with reference to the research of Balli and Sørensen (2013) [64], for the interaction term (*FUND×DZ-score*), FUND is decentralized according to the industry-annual standard, and is represented by DFUND.

**Table 10:** Regression results for model 3.

Variable	(1)	(2)	(3)	(4)
	Coef. (S.E.)	Coef. (S.E.)	Coef. (S.E.)	Coef. (S.E.)
Intercept	-2.977***(0.669)	-2.942***(0.670)	-7.762***(0.817)	-7.748***(0.818)
FUND	-0.008**(0.003)	-0.007**(0.003)	-0.011***(0.004)	-0.009**(0.004)
DZ-score	0.115*(0.063)	0.025(0.057)	-0.007(0.065)	-0.090(0.058)
FUND×DZ-score	-0.015*(0.007)		-0.012*(0.007)	
DFUND×DZ-score		-0.017**(0.007)		-0.019***(0.007)
L.ROA	-0.005(0.004)	-0.005(0.004)	-0.002(0.004)	-0.002(0.004)
L.TAT	0.548***(0.044)	0.548***(0.044)	0.459***(0.045)	0.460***(0.045)
L.LEV	-0.016***(0.002)	-0.016***(0.002)	-0.014***(0.002)	-0.014***(0.002)
L.GROWTH	-0.068(0.082)	-0.066(0.082)	-0.151*(0.090)	-0.149*(0.090)
ShrZ	0.028(0.141)	0.015(0.141)	0.035(0.121)	0.019(0.121)
AGE	0.269***(0.004)	0.269***(0.004)	0.257***(0.004)	0.257***(0.004)
IN_DIREC	0.069(0.344)	0.054(0.344)	0.461(0.365)	0.437(0.365)
LnASSET	0.409***(0.022)	0.408***(0.022)	0.383***(0.023)	0.382***(0.023)
L.LnSALARY	-0.204***(0.032)	-0.204***(0.032)	-0.203***(0.033)	-0.202***(0.033)
L.AUDIT	-0.818***(0.295)	-0.816***(0.295)	-0.751**(0.337)	-0.746**(0.337)
STATE	0.044(0.046)	0.045(0.046)	-0.001(0.048)	-0.001(0.048)
YEAR/IND	YES	YES	YES	YES
# of obs.	3594	3594	2802	2802
Adj_R^2^	0.707	0.707	0.671	0.672
F_Value	364.71***	249.84***	249.84***	249.54***

Note

*** Significant at 1%

** Significant at 5%

* Significant at 10%.

Robust standard errors in brackets are clustered at corporate level.

As shown in Table 10, from columns 1 to 4, the coefficients on FUND are negative and significant (-0.008, *p*< 0.05; -0.007, *p*< 0.05; -0.011, *p*< 0.01; -0.009, *p*< 0.05). The coefficients on FUND×DZ-score are negative and significant (-0.015, *p*< 0.10; -0.012, *p*< 0.10). And those on DFUND×DZ-score are negative and significant (-0.017, *p*< 0.05; -0.019, *p*< 0.01). Again, the results show that fund shareholding can inhibit the lack of R&D input, and these show a higher level of inhibiting effect when the risk of financial failure is higher. Hypotheses 1 and 2 above are verified again. Besides, the conclusions on the control variables are consistent with those from Table 7 or Table 8.

## 7. Further analysis

According to the above analyses, fund shareholding has an inhibiting effect on the insufficient R&D input. Meanwhile, the previous literature has shown that institutional investors are influenced by the geographical characteristics of listed companies when making shareholding decisions, and their shareholding ratio in listed companies in eastern cities is higher than that in central and western cities [15]. Moreover, in accordance with the investigation and research of Wang et al. (2019) [65], there are great differences in the institutional environment such as economic development and marketization degree among the provinces and regions in China. The process of regional marketization affects the shareholding ratio of fund companies. In the regions with a higher degree of marketization, the government is more open-minded and takes less intervention in the market. Enterprises are more willing to make profits through the continuous R&D input and improvement of innovation efficiency. Then, will the marketization process significantly affect the relationship between fund shareholding and insufficient R&D input? This study expects that institutional constraints can effectively alleviate the information asymmetry in a perfect market environment, improve the willingness of fund companies to hold shares, and then strengthen the inhibiting effect of fund shareholding on insufficient R&D input. To verify this moderating effect, the following model 4 is constructed for empirical test. With reference to the research of Li and Yan (2019) [16], in model 4, the value of marketization level (MARKET) is derived from the “Marketization Index of China’s Provinces: Neri Report 2018” [65]. Besides, to alleviate the adverse effect of multicollinearity on the analyses, with reference to the research of Balli and Sørensen (2013) [64], for the interaction term (DFUND×DMARKET), FUND is decentralized according to the industry-annual standard, and is represented by DFUND. Meanwhile, MARKET is decentralized according to the annual standard, and is represented by DMARKET. Table 11 reports the regression results for model 4.

**Table 11:** Regression results for model 4.

Variable	(1)	(2)	(3)	(4)
	Coef. (S.E.)	Coef. (S.E.)	Coef. (S.E.)	Coef. (S.E.)
Intercept	-6.146*** (0.700)	-5.978*** (0.709)	-7.284***(0.819)	-7.247***(0.818)
FUND	-0.011*** (0.003)	-0.012*** (0.003)	-0.013***(0.003)	-0.015***(0.003)
MARKET	-0.072*** (0.011)	-0.077*** (0.011)	-0.059***(0.011)	-0.062***(0.011)
DFUND×DMARKET		-0.004** (0.002)		-0.003*(0.002)
L.ROA	-0.007* (0.004)	-0.006 (0.004)	-0.002 (0.004)	-0.002 (0.004)
L.TAT	0.560*** (0.041)	0.560*** (0.041)	0.500***(0.043)	0.498***(0.043)
L.LEV	-0.016*** (0.001)	-0.016*** (0.001)	-0.015***(0.001)	-0.015***(0.001)
L.GROWTH	-0.083 (0.082)	-0.085 (0.082)	-0.016*(0.009)	-0.016*(0.009)
ShrZ	0.010 (0.139)	0.018 (0.139)	0.017 (0.121)	0.021 (0.120)
AGE	0.267*** (0.004)	0.267*** (0.004)	0.257***(0.004)	0.256***(0.004)
IN_DIREC	0.046 (0.343)	0.039 (0.343)	0.454(0.365)	0.449 (0.365)
LnASSET	0.407*** (0.022)	0.408*** (0.022)	0.372***(0.022)	0.372***(0.022)
L.LnSALARY	-0.171*** (0.033)	-0.171*** (0.033)	-0.173***(0.033)	-0.173***(0.033)
L.AUDIT	-0.814*** (0.295)	-0.827*** (0.295)	-0.766**(0.337)	-0.771**(0.337)
STATE	0.014 (0.047)	0.019 (0.047)	-0.019 (0.048)	-0.014 (0.048)
YEAR/IND	YES	YES	YES	YES
# of obs.	3594	3594	2802	2802
Adj_R^2^	0.710	0.711	0.674	0.674
F_Value	381.79***	369.44***	259.86***	252.05***

Note

*** Significant at 1%

** Significant at 5%

* Significant at 10%.

Robust standard errors in brackets are clustered at corporate level.

Model 4.

$${\mathrm{UnderR}\&D}_{i,t}=\zeta_{0}+\zeta_{1}{\mathrm{FUND}}_{i,t}+\zeta_{2}{\mathrm{MARKET}}_{i,t}+\zeta_{3}{\mathrm{DFUND}}_{i,t}\times {\mathrm{DMARKET}}_{i,t}+\zeta_{4}{\mathrm{ROA}}_{i,t\mathrm{-1}}+\zeta_{5}{\mathrm{TAT}}_{i,t‐1}+\zeta_{6}{\mathrm{LEV}}_{i,t‐1}+\zeta_{7}{\mathrm{GROWTH}}_{i,t‐1}+\zeta_{8}{\mathrm{ShrZ}}_{i,t}+\zeta_{9}{\mathrm{AGE}}_{i,t}+\zeta_{10}\mathrm{IN}\_{\mathrm{DIREC}}_{i,t}+\zeta_{11}{\mathrm{LnASSET}}_{i,t}+\zeta_{12}\mathrm{LnSALAR}Y_{i,t‐1}+\zeta_{13}{\mathrm{AUDIT}}_{i,t‐1}+\zeta_{14}{\mathrm{STATE}}_{i,t}+\zeta_{15}\sum_{t}\mathrm{YEAR}+\zeta_{16}\sum_{t}\mathrm{IND}+\varepsilon_{i,t}$$ (5)

In Table 11, columns 1 and 3 report the results without interaction item, and columns 2 and 4 report the results with interaction item. Moreover, columns 3 and 4 show the results after re-estimating UnderR&D (*In section 6*.*1*). The coefficients on MARKET are negative and significant (-0.072, *p* < 0.01; -0.077, *p* < 0.01; -0.059, *p* < 0.01; -0.062, *p* < 0.01), indicating that the higher the degree of marketization in the regions where the enterprise is located, the more fair and orderly the market competition will be, which can promote the more transparent and open decision-making environment of R&D investment. A good market environment is conducive to reducing transaction costs, thus improving the intensity of R&D input and alleviating the insufficient R&D input. In columns2 and 4, the coefficients on DFUND×DMARKET are negative and significant (-0.004, *p* < 0.05; -0.003, *p* < 0.10), implying that the marketization process has a significant moderating effect between fund shareholding and insufficient R&D input. A higher level of marketization is conducive to strengthening the inhibiting effect of fund shareholding on insufficient R&D input, and stimulating enterprises’ innovation activities. Therefore, the enterprises in these regions are encouraged to gain profits by increasing R&D input and improving innovation efficiency. These results suggest that the regulators should improve the marketization process in the regions with lower marketization, change the status quo that the government is relatively conservative and intervenes more in the market, and promote the market to gradually transform to an orderly competition order.

In the control variables, the conclusions on L.TAT, L.LEV, AGE, LnASSET, L.LnSALARY and L.AUDIT are consistent with those from Table 7. And in columns 3 and 4, the conclusion on L. GROWTH is consistent with that from Table 8. Besides, in column 1, the coefficient on L.ROA is negative and significant (-0.007, *p*< 0.10). The possible reason is that enterprises’ good accounting surplus provides material guarantee for R&D activities and alleviates the possible insufficient R&D input under the premise of normal operation.

## 8. Conclusions and recommendations

### 8.1 Conclusions

Based on the principal-agent theory and the financial management theory, this study empirically tests the impact of fund shareholding on insufficient R&D input, and explores the mechanism of fund shareholding on corporate innovation. The results show that with the increase of fund shareholding, it can promote the power and ability of fund investors to influence corporate R&D activities, and inhibit the situation of insufficient R&D input. This result do not support the conclusion of Wen and Feng (2012) [9], Lai and Sun (2017) [10] that securities investment funds have a negative impact on corporate innovation. Different from the existing literatures that take the number of patents as the proxy of innovation activities [12, 13], this study enriches the research horizons on corporate innovation from the perspective of insufficient R&D input, and enriches the literatures on the economic effect of fund shareholding. Meanwhile, the inhibiting effect of fund shareholding on insufficient R&D input is mainly reflected in the case of higher risk of financial failure. It is an important mechanism for fund shareholding to promote corporate innovation to alleviate insufficient R&D input when the risk of financial failure is higher. On basis of distinguishing the risk levels of financial failure, this study explores the mechanism of fund shareholding on innovation activities, and makes up for the shortage that few literatures have included fund shareholding, R&D input and financial failure risk into the same framework. The further analyses show that the higher marketization strengthens the inhibiting effect of fund shareholding on insufficient R&D input, and has a stimulating effect on corporate innovation activities. This study provides evidence support for the regulatory authorities to promote the process of marketization, promote fund companies to participate in corporate governance to enhance enterprises’ competitiveness. Securities investment funds increase their holdings and actively promote corporate technological innovation.

### 8.2 Recommendations

At present, the “mass entrepreneurship and innovation” should be promoted, to stimulate the enthusiasm and potential of innovation entities. Enterprises should make more information about R&D plans and decision-making transparent, and communicate with institutional investors such as fund companies, so that they can obtain more information about enterprises’ long-term value and make more valuable investments. Fund companies are encouraged to hold listed companies’shares, and their proposal power in the stockholders’ meeting is appropriately enhanced. Fund companies participate in corporate governance to avoid the managers’ short-sighted and opportunistic behaviors, to improve the efficiency of R&D activities, especially to avoid insufficient R&D input when the risk of financial crisis is higher, and then improve enterprises’ independent innovation ability.

Enterprises are the main body of innovation system. It will be the focus of the real economy in the future to realize the transformation of enterprises from “following the lead” to “running in parallel” and “leading the way”. The regulators continue to regulate the market operation, promote the development of securities investment funds, and guide them to play an active role in external governance. Fund companies establish a sense of long-term investment, realize the effective supervision to inhibit insufficient R&D input, and promote the capital market’s benign development. In addition to the continued improvement of the regulatory system, it is necessary to reduce the threshold of securities investment funds to enter the market, to play their active governance role and enhance the competitiveness in the industry. Meanwhile, the regulators constantly promote the market-oriented reform and optimize the regional institutional environment. It is necessary to promote the process of regional marketization, to strengthen the inhibiting effect of fund shareholding on insufficient R&D input, improve the efficiency of R&D input and play a promoting role in innovation activities.

### 8.3 Limitations and prospects

The intensity of R&D input represents the importance enterprises attach to innovation activities. Enterprises’ innovation activities are characterized by the strong professionalism, high confidentiality and great uncertainty, so the growth of R&D input is not equal to the improvement of innovation performance [66]. Ultimately, the improvement of innovation performance is mainly reflected by the results of innovation input, such as the increase of new product sales, patent application or grant, etc. Due to the limited space, this study does not combine fund shareholding, R&D input and ultimate innovation performance. Meanwhile, internal control, an important part of corporate governance, is not considered in this paper. The internal control ensures reasonably the improvement of operating efficiency and effect, and promotes the realization of the development strategy of enterprises. In the future research, fund shareholding as an external governance element, internal control as an internal governance element, R&D input and actual innovation results may be included into the same research framework. It is expected to expound the joint action mechanism of fund shareholding and internal control in the process of transforming R&D input into innovation achievements, which may be a major research prospect in the future.

## Supporting information

S1 Data The data set used in this article for discussion and analysis. (ZIP)
