# Supplementary material for: Can fund shareholding inhibit insufficient R&D input?——Empirical evidence from Chinese listed companies
Source: PLoS One. 2021 Mar 25;16(3):e0248674. doi: 10.1371/journal.pone.0248674 (PMC7993821; doi:10.1371/journal.pone.0248674)
Supplement: S1 Data — (ZIP) [file pone.0248674.s001.zip › S1/Estimation of UnderR&D/Code_UnderR&D.docx]

(1) System GMM

xtdpdsys RD L.Growth L.LEV L.CASH L.AGE L.LnASSET dum*, lags(1) twostep vce (robust)

xtdpdsys RD L.Growth L.LEV L.CASH L.AGE L.LnASSET dum_yr3-dum_yr6 dum_ind1-dum_ind7 dum_ind9-dum_ind13 dum_ind15 dum_ind16, lags(1) twostep vce (robust)

estat abond

xtdpdsys RD L.Growth L.LEV L.CASH L.AGE L.LnASSET dum_yr3-dum_yr6 dum_ind1-dum_ind7 dum_ind9-dum_ind13 dum_ind15 dum_ind16, lags(1) twostep

estat sargan

(2) Fixed effects model

Xtreg RD L.Growth L.LEV L.CASH L.AGE L.LnASSET L.RD dum*, fe r

predict RD1

gen e1=RD - RD1

gen ABSIE= abs(e1)

gen ABSIEO=e1 if e1>0

gen UnderRD=abs(e1) if e1<0

bysort ind year:egen M1=pctile(ABSIE),p(20)

bysort ind year:egen M2=pctile(ABSIEO),p(20)

bysort ind year:egen M3=pctile(UnderRD),p(20)

xtset code1 year
