# Supplementary material for: Can fund shareholding inhibit insufficient R&D input?——Empirical evidence from Chinese listed companies
Source: PLoS One. 2021 Mar 25;16(3):e0248674. doi: 10.1371/journal.pone.0248674 (PMC7993821; doi:10.1371/journal.pone.0248674)
Supplement: S1 Data — (ZIP) [file pone.0248674.s001.zip › S1/Robust_Test_1/Code_Robust_Test_1.docx]

**Robustness test 1**

**(1) Reestimate the explained variable**

regress UnderRD FUND L.ROA L.TAT L.LEV L.GROWTH ShrZ AGE IN_DIRECTOR L.LnASSET L.LnSALARY L.AUDIT STATE dum* if UnderRD >M3, r

regress UnderRD FUND L.ROA L.TAT L.LEV L.GROWTH ShrZ AGE IN_DIRECTOR L.LnASSET L.LnSALARY L.AUDIT STATE dum* if Z>2.675& UnderRD >M3, r

regress UnderRD FUND L.ROA L.TAT L.LEV L.GROWTH ShrZ AGE IN_DIRECTOR L.LnASSET L.LnSALARY L.AUDIT STATE dum* if Z<=2.675& UnderRD>M3, r

keep if UnderRD >M3

global xx "FUND ROA1 TAT1 LEV1 GROWTH1 ShrZ AGE IN_DIRECTOR LnASSET1 LnSALARY1 AUDIT1 STATE dum*"

bdiff, group(RISK) model(regress UnderRD $xx, r) reps(100) detail
