# Supplementary material for: Can fund shareholding inhibit insufficient R&D input?——Empirical evidence from Chinese listed companies
Source: PLoS One. 2021 Mar 25;16(3):e0248674. doi: 10.1371/journal.pone.0248674 (PMC7993821; doi:10.1371/journal.pone.0248674)
Supplement: S1 Data — (ZIP) [file pone.0248674.s001.zip › S1/Robust_Test_2/Code_Robust_Test_2.docx]

**Robust Test 2.**

**(2) Instrumental variable method**

ivregress 2sls UnderRD L.ROA L.TAT L.LEV L.GROWTH ShrZ AGE IN_DIRECTOR L.LnASSET L.LnSALARY L.AUDIT STATE dum* (FUND=L.FUND Num_FUND) , r first

ivregress 2sls UnderRD L.ROA L.TAT L.LEV L.GROWTH ShrZ AGE IN_DIRECTOR L.LnASSET L.LnSALARY L.AUDIT STATE dum* (FUND=L.FUND Num_FUND) if Z>2.675, r first

ivregress 2sls UnderRD L.ROA L.TAT L.LEV L.GROWTH ShrZ AGE IN_DIRECTOR L.LnASSET L.LnSALARY L.AUDIT STATE dum* (FUND=L.FUND Num_FUND) if Z<=2.675, r first
