# Supplementary material for: Can fund shareholding inhibit insufficient R&D input?——Empirical evidence from Chinese listed companies
Source: PLoS One. 2021 Mar 25;16(3):e0248674. doi: 10.1371/journal.pone.0248674 (PMC7993821; doi:10.1371/journal.pone.0248674)
Supplement: S1 Data — (ZIP) [file pone.0248674.s001.zip › S1/Robust_Test_3/Code_Robust_Test_3.docx]

**Robust Test 3.**

**(3) Change model 2**

① Interaction item is not centralized

regress UnderRD FUND DZ F_DZ L.ROA L.TAT L.LEV L.GROWTH ShrZ AGE IN_DIRECTOR L.LnASSET L.LnSALARY L.AUDIT STATE dum*, r

regress UnderRD FUND DZ F_DZ L.ROA L.TAT L.LEV L.GROWTH ShrZ AGE IN_DIRECTOR L.LnASSET L.LnSALARY L.AUDIT STATE dum* if UnderRD>M3, r

gen DZ =(Z<=2.675)

gen F_DZ=FUND*DZ

xtset code1 year

② Interaction item is centralized

regress UnderRD FUND DZ DF_DZ L.ROA L.TAT L.LEV L.GROWTH ShrZ AGE IN_DIRECTOR L.LnASSET L.LnSALARY L.AUDIT STATE dum*, r

regress UnderRD FUND DZ DF_DZ L.ROA L.TAT L.LEV L.GROWTH ShrZ AGE IN_DIRECTOR L.LnASSET L.LnSALARY L.AUDIT STATE dum* if UnderRD>M3, r

xtset code1 year

bysort ind year: egen MFUND =mean(FUND)

xtset code1 year

xtset code1 year

gen DFUND = FUND - MFUND

xtset code1 year

gen DF_DZ=DFUND*DZ

xtset code1 year
