# Supplementary material for: Can fund shareholding inhibit insufficient R&D input?——Empirical evidence from Chinese listed companies
Source: PLoS One. 2021 Mar 25;16(3):e0248674. doi: 10.1371/journal.pone.0248674 (PMC7993821; doi:10.1371/journal.pone.0248674)
Supplement: S1 Data — (ZIP) [file pone.0248674.s001.zip › S1/Main Regress and Further Analysis/Code_Main Regress and Further Analysis.doc]

**1. Descriptive statistics**

tabstat UnderRD FUND ROA TAT LEV GROWTH ShrZ AGE IN_DIRECTOR LnASSET LnSALARY AUDIT STATE, stat(mean median max min sd count) col(stat)

**2. Univariate analysis**

ttable3 UnderRD FUND ROA TAT LEV GROWTH ShrZ AGE IN_DIRECTOR LnASSET LnSALARY AUDIT STATE, by(RISK)

ttable3 UnderRD FUND ROA TAT LEV GROWTH ShrZ AGE IN_DIRECTOR LnASSET LnSALARY AUDIT STATE, by(RISK) median

**3. Variable correlation**

pwcorr UnderRD FUND ROA TAT LEV GROWTH ShrZ AGE IN_DIRECTOR LnASSET LnSALARY AUDIT STATE, sig star(0.1)

**4. Main regress**

(1) regress UnderRD FUND L.ROA L.TAT L.LEV L.GROWTH ShrZ AGE IN_DIRECTOR L.LnASSET L.LnSALARY L.AUDIT STATE dum*, r

(2) regress UnderRD FUND L.ROA L.TAT L.LEV L.GROWTH ShrZ AGE IN_DIRECTOR L.LnASSET L.LnSALARY L.AUDIT STATE dum* if Z>2.675, r

(3) regress UnderRD FUND L.ROA L.TAT L.LEV L.GROWTH ShrZ AGE IN_DIRECTOR L.LnASSET L.LnSALARY L.AUDIT STATE dum* if Z<=2.675, r

(4) *Fisher’s Permutation test*

global xx "FUND ROA1 TAT1 LEV1 GROWTH1 ShrZ AGE IN_DIRECTOR LnASSET1 LnSALARY1 AUDIT1 STATE dum*"

bdiff, group(RISK) model(regress UnderRD $xx, r) reps(100) detail

**5. Further Analysis**

(1) regress UnderRD FUND MARKET L.ROA L.TAT L.LEV L.GROWTH ShrZ AGE IN_DIRECTOR L.LnASSET L.LnSALARY L.AUDIT STATE dum*, r

regress UnderRD FUND MARKET DF L.ROA L.TAT L.LEV L.GROWTH ShrZ AGE IN_DIRECTOR L.LnASSET L.LnSALARY L.AUDIT STATE dum*, r

(2) regress UnderRD FUND MARKET L.ROA L.TAT L.LEV L.GROWTH ShrZ AGE IN_DIRECTOR L.LnASSET L.LnSALARY L.AUDIT STATE dum* if UnderRD>M3, r

regress UnderRD FUND MARKET DF L.ROA L.TAT L.LEV L.GROWTH ShrZ AGE IN_DIRECTOR L.LnASSET L.LnSALARY L.AUDIT STATE dum* if UnderRD>M3, r
